# Panicle-3D: Efficient Phenotyping Tool for Precise Semantic Segmentation of Rice Panicle Point Cloud

**DOI:** 10.34133/2021/9838929

**Published:** 2021-12-23

**Authors:** Liang Gong, Xiaofeng Du, Kai Zhu, Ke Lin, Qiaojun Lou, Zheng Yuan, Guoqiang Huang, Chengliang Liu

**Affiliations:** ^1^School of Mechanical Engineering, Shanghai Jiao Tong University, Shanghai 200240, China; ^2^Shanghai Agrobiological Gene Center, Shanghai 201106, China; ^3^Centre for Agriculture and Health, School of Life Sciences and Biotechnology, Shanghai Jiao Tong University, Shanghai 20040, China

## Abstract

The automated measurement of crop phenotypic parameters is of great significance to the quantitative study of crop growth. The segmentation and classification of crop point cloud help to realize the automation of crop phenotypic parameter measurement. At present, crop spike-shaped point cloud segmentation has problems such as fewer samples, uneven distribution of point clouds, occlusion of stem and spike, disorderly arrangement of point clouds, and lack of targeted network models. The traditional clustering method can realize the segmentation of the plant organ point cloud with relatively independent spatial location, but the accuracy is not acceptable. This paper first builds a desktop-level point cloud scanning apparatus based on a structured-light projection module to facilitate the point cloud acquisition process. Then, the rice ear point cloud was collected, and the rice ear point cloud data set was made. In addition, data argumentation is used to improve sample utilization efficiency and training accuracy. Finally, a 3D point cloud convolutional neural network model called Panicle-3D was designed to achieve better segmentation accuracy. Specifically, the design of Panicle-3D is aimed at the multiscale characteristics of plant organs, combined with the structure of PointConv and long and short jumps, which accelerates the convergence speed of the network and reduces the loss of features in the process of point cloud downsampling. After comparison experiments, the segmentation accuracy of Panicle-3D reaches 93.4%, which is higher than PointNet. Panicle-3D is suitable for other similar crop point cloud segmentation tasks.

## 1. Introduction

The automated measurement of crop phenotypic parameters is of great significance for studying the effects of crop genes and growth environment on crop phenotypes. For example, parameters such as rice stem diameter and ear length are related to the lodging resistance and yield of rice [1–3]. Traditional 3D point cloud segmentation methods include edge-based segmentation algorithms, region-based segmentation algorithms (such as region growing algorithms [4]), attribute-based segmentation algorithms, and image segmentation-based segmentation algorithms. Although these algorithms can be migrated to plant point cloud segmentation, due to the complexity of plant point cloud morphology, targeted improvements are often required to achieve better segmentation results. Besides, the individual plants of the same variety differ greatly in morphology, and the insufficient generalization performance of the above algorithm makes the segmentation effect often not stable enough.

In recent years, deep learning has achieved rapid development with the growth of algorithm theory and hardware computing power. Especially with the introduction of convolutional neural networks and fully convolutional neural networks [5], deep learning algorithms have solved many problems that traditional algorithms are difficult to handle effectively. Especially in the image field, the effects of deep learning on data classification, segmentation, and recognition and positioning greatly exceed traditional algorithms. Although related issues in the biological field are more complex, deep learning has also achieved good results [6]. For example, Pound et al. used CNN for root structure analysis [7]; the U-net network model proposed by Olaf Ronneberger et al. used very few pictures for end-to-end training and achieved good results in biological image segmentation problems.

However, there are few related studies and applications of deep learning in the analysis of 3D models of plants. One problem is that the relevant 3D data sets of plants are difficult to obtain, and the complicated structure makes data labeling require a lot of manpower [8]. Existing common open-source data sets used for point cloud segmentation include: large-scale outdoor remote sensing 3D data set http://Semantic3D.net [9], which contains scene data such as outdoor buildings, roads, and trees; Stanford large-scale indoor RGB-D data S3DIS [10], including various objects in indoor scenes; ModelNet40 [11] is a point cloud including tables, chairs, airplanes, and cars for point cloud classification and segmentation. In addition, there are 3D datasets for other scenes, but there is a lack of available plant segmentation datasets. Another problem is that the data form of the 3D point cloud has disorder and rotation invariance as non-Euclidean data, and it is difficult to apply general convolutional neural networks to scattered and disordered point clouds.

One solution is to divide the point cloud by voxels and use CNN for semantic segmentation [12], but this will bring a lot of calculation and memory requirements [13]. To improve memory utilization efficiency and calculation speed, Wang et al. used octrees to divide voxels for optimization [14] and achieved good segmentation results.

The PointNet series of neural networks pioneered an end-to-end semantic segmentation method [15], which greatly improved the accuracy of the algorithm on related data sets. The core idea is to use the symmetric method to solve the disorder of the point cloud and to use the STN network [16] to achieve rotation invariance. Related improved algorithms based on PointNet have also refreshed the point cloud segmentation rankings. For example, PointSIFT [17] of the Lu Cewu team of Shanghai Jiao Tong University added the point cloud 3D SIFT feature to improve the effectiveness of the model. Deep learning models based on the PointNet series are usually based on the feature extraction capabilities of the enhanced model, especially the local features and relationships of points. Wang et al. believe that the loss of the relationship between local features and local points is still a factor that limits model performance [18]. To overcome this problem, a dynamic graph convolutional neural network (DGCNN) is proposed, in which the EdgeConv structure can extract edge features while keeping the ordering unchanged. Inspired by the idea of an attention mechanism, Wang et al. [19] designed a graph attention convolution (GAC) in which the kernel can dynamically adapt to the structure of the object. GAC can capture the structural features of the point cloud while avoiding feature pollution between targets.

In this study, we first used a special turntable-based structured light 3D scanning platform to scan rice ears to obtain 3D point cloud data of rice ears. Then, use the open source software *labelme* to mark the point cloud point by point and create a rice ear point cloud data set. Finally, a 3D point cloud convolutional neural network based on the PointConv module is designed, and the abovementioned data set is used for training to realize the effective segmentation of rice ears and stalks.

## 2. Materials and Methods

### 2.1. Data Acquisition

As a high-precision, high-efficiency noncontact measurement method, optical three-dimensional scanning technology has been widely used in many fields such as industrial automation, cultural relic restoration, and reverse engineering [20]. To easily obtain 3D point cloud data of rice and analyze its phenotypic parameters, we built an automated multiview point cloud scanning platform based on Digital Light Processing (DLP), rotating platform, and USB camera and developed point cloud stitching software based on Point Cloud Lib (PCL) and OpenCV.

**Figure 1:**
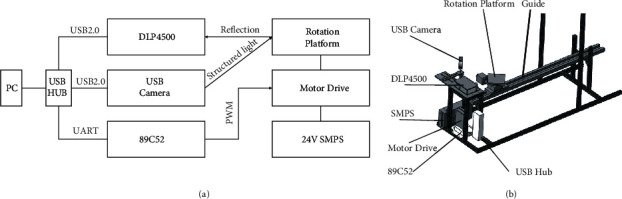
The structure of the point cloud acquisition platform.

DLP technology is widely used in optical engineering due to its outstanding performance. The core component of our scanning device is the Texas Instruments DLP4500 evaluation module. Texas Instruments (TI) provides an accurate point cloud generation open-source software development kit based on DLP4500. However, it can only be used to collect point cloud information in one direction of the object and cannot meet the needs of the overall 3D reconstruction of complex parts. The automatic rotating platform controlled by Microcontroller Unit solves this problem well. During the acquisition of the 3D point cloud, the DLP4500 evaluation module and the automatic rotating platform will alternately complete the scanning and rotating processes until all the surface information of the scanned object is collected. The structure of the platform is shown in Figure 1.

### 2.2. Data Preprocessing

**Figure 2:**
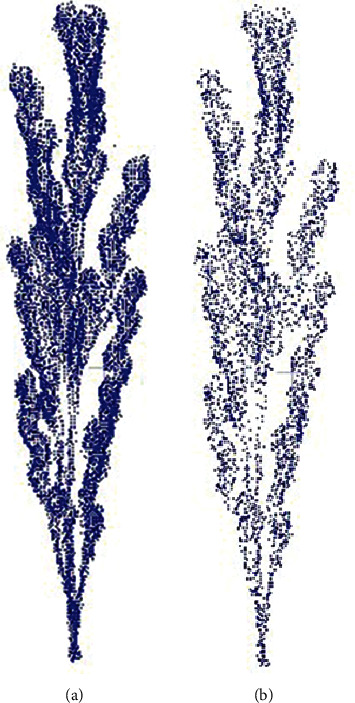
(a) Point cloud before downsampling. (b) Point cloud after downsampling.

We first manually remove outliers from the point cloud collected by the device and then use the point cloud Gaussian filtering algorithm to remove the noise points in the point cloud. Finally, in order to make the number of points of a single point cloud meet the input layer size of the neural network (2048 points), we use the farthest point sampling algorithm to downsample the point cloud. The farthest point sampling algorithm can ensure that the point cloud is downsampled uniformly. A sampling result is shown in Figure 2.

**Figure 3:**
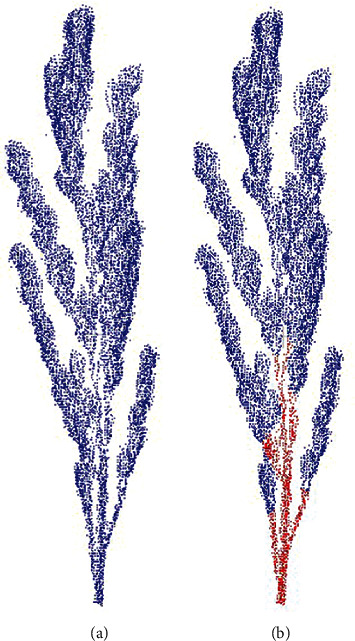
(a) Point cloud before being labeled. (b) Point cloud after being labeled;

Point cloud labeling is labor-intensive. Two-dimensional image annotation relies on many existing tools, such as *labelme* [21]. There are also related tools for 3D point cloud annotation, such as the shortest path tree-based segmentation algorithm developed by Monica et al. [22]. Yan et al. [23] developed a semiautomatic point cloud annotation tool based on point cloud clustering. In this paper, CloudCompare [24] is used to perform point cloud-assisted segmentation and then annotated to generate a data set. As shown in Figure 3, the point cloud is finally marked as two parts of rice stalk (red) and rice ear (blue).

**Figure 4:**
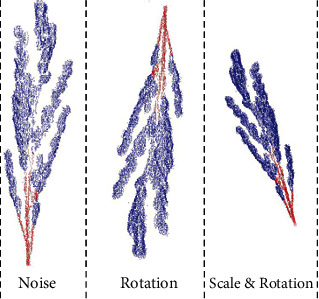
Rice point cloud data argumentation.

A good data set is a prerequisite for training an excellent neural network. If the data set is too small, it will easily lead to overfitting of the model, and the quantity and diversity of data will affect the performance of the model [25]. Because the data set of the 3D point cloud is too complicated to make, the amount of data is inevitably limited. Therefore, a series of operations such as rotation, scaling, cropping, and Gaussian noise increase is needed to achieve the purpose of data enhancement, as shown in Figure 4.

### 2.3. Panicle-3D Model Architecture

In the point cloud segmentation task, the output of the neural network should be the prediction result of each point. To obtain high-level semantic information, point cloud data is downsampled in the forward propagation process and should be upsampled layer by layer during output to form an encoding-decoding structure.

Based on the above framework, it is natural to embed a convolution suitable for 3D point clouds. However, most 3D convolution schemes are implemented by converting to 2D images or voxels, and these methods are very computationally intensive. To reduce the amount of computation and memory usage, this paper adopts the Point Conv (PointConv) scheme proposed by Wu et al. [26].

For discrete point cloud, 3D convolution (PointConv) is defined as:

(1) $$\begin{array}{c} \text{PointConv}\left(S,W,F\right)=\underset{\left(\delta_{x},\delta_{y},\delta_{z}\right)\in G}{\sum }S\left(\delta_{x},\delta_{y},\delta_{z}\right)W\left(\delta_{x},\delta_{y},\delta_{z}\right)F\left(x+\delta_{x},y+\delta_{y},z+\delta_{z}\right). \end{array}$$

*G* represents the local neighborhood of the point *P* (*x*, *y*, *z*). *F*(*x* + *δ*_*x*_, *y* + *δ*_*y*_, *z* + *δ*_*z*_) represents the feature of the neighborhood point. The neighborhood is defined by the *k*-nn algorithm, and (*δ*x, *δ*y, *δ*z) represents the differential of the three-dimensional position in space. *S* is the inverse density at point Delta. *W* is the weight function approximated by PointConv.

This kind of convolution uses two multilayer perceptrons (MLP) to learn the continuous convolution weight function [27] and the inverse density weight function [26]. Similarly, this paper adopts the point cloud deconvolution scheme proposed by Wu et al. [26]. The point cloud deconvolution layer is realized by using the low-level dense point cloud as the upsampling input, while using the high-level features to perform linear interpolation on it and then connecting a PointConv layer.

Drozdzal et al. studied the influence of both long and short skip connections on fully convolutional networks (FCN) for biomedical image segmentation [28]. This research also introduces the long-short jump method to optimize the model.

To improve the accuracy of point cloud segmentation, the model designed in this paper adopts a crosslayer connection structure. The crosslayer connection structure is a structure proposed by Shelhamer et al. for image pixel-level semantic segmentation tasks [5]. This structure connects the low-level features of the encoder with the high-level features of the decoder, effectively fusing high-level and low-level semantic information [29]. When performing deconvolution, the structure of crosslayer connection enables low-level semantic information to be used for the segmentation of point cloud details. Neural networks with similar structures such as U-Net also use this crosslayer connection structure, which has achieved very good results in the field of biological images [30]. After downsampling and upsampling, the number of output point cloud points is the same as the number of input point cloud points, and the point cloud is divided point by point. These structures help the neural network to combine information of different scales and improve the accuracy of the edges.

**Figure 5:**
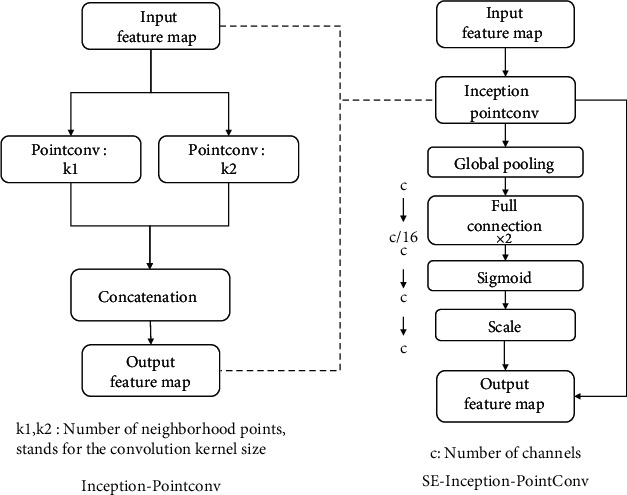
The structure of the SE-Net module and the structure of the SE-Inception-PointConv module.

To improve the performance of the model and adapt it to the segmentation task of rice ear point cloud, this paper constructs the SE-Inception-PointConv structure, embedded inception structure [31], and SE-Net structure (squeeze-and-excitation net) in the original PointConv structure [32]. On the one hand, the Inception structure increases the width of the network, and on the other hand, increases the network's adaptability to scale. SE-Net was proposed by Hu et al. [33]. By training SE-Net and recalibrating the response coefficients of different channel characteristics, the interdependence between channels can be found. This structure can significantly improve the performance of the neural network without increasing the training parameters. Furthermore, SE-Net can be combined with the Inception structure and then embedded in the PointConv convolutional layer.  Figure 5 shows the structure of the SE-Net module and the structure of the SE-Inception-PointConv module used in this article.

As shown in the figure above, the ReLU activation function [34] is added between the entrance of each module and the two fully connected layers of the SE module, and the channel compression factor is set to 4. The practice has proved that SE-Inception-PointConv can significantly improve the performance of CNN with minimal additional computational cost. At the same time, compared with the total parameter amount of CNN, the increase of the weight parameter amount is negligible.

**Figure 6:**
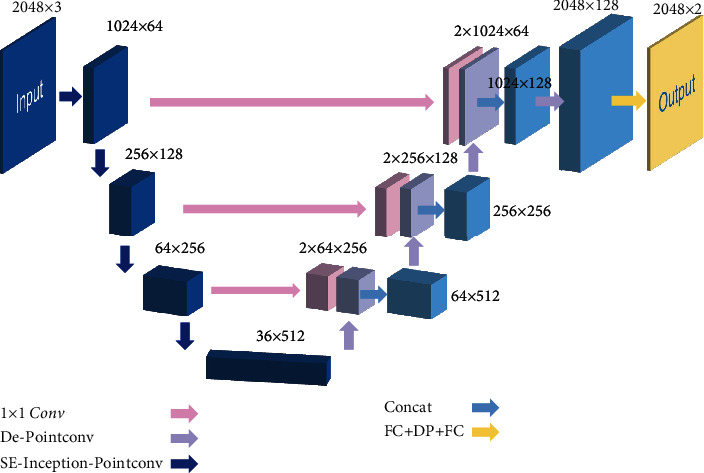
Structure of Panicle-3D.

Based on the above SE-Inception-PointConv module, this paper designs the Panicle-3D neural network as shown in Figure 6. The Panicle-3D network is a U-Net-like FCN architecture, and there are long skip connections from contracting path to expanding path. The blocks like De-PointConv and SE-Inception-PointConv contain short skip connections [28].

The encoding path contains 4 neural network blocks. Except for the input layer and the maximum pooling layer, each block is composed of the aforementioned SE-Inception-PointConv module and is downsampled. The neighborhood size k1 and k2 of the PointConv convolution layer (similar to the size of the convolution kernel) are 16 and 32, respectively. Correspondingly, the decoding path has a deconvolution layer, which is connected in cascade with the output copy of the previous layer, and a 1 × 1 convolution layer is used to fuse channel information. During the encoding process, the number of feature channels is increased from 3 to 512, and the number of points in the point cloud is reduced from 2048 points to 36 points. During the decoding process, the number of feature channels is reduced from 512 to 128, and the number of point cloud points is increased from 36 to 2048 pixels. To avoid overfitting, a dropout layer is added before the output layer [35].

The output layer of the model is a 1 × 1 convolutional layer, which outputs two channels. The output layer is activated by the sigmoid function. The value of each point represents the probability. Without loss of generality, if the predicted probability value is greater than 0.5, it is marked as the corresponding label.

### 2.4. Model Training Methods

The above model is optimized using the crossentropy loss (cost) function [36], as shown in formula (2), *y*_*i*_ represents the true label of the point cloud point, and *p*_*i*_ represents the predicted value calculated by the model.

(2) $$\begin{array}{c} Loss=\overset{m}{\underset{i=1}{\sum }}-\left(y_{i}\log\left(p_{i}\right)+\left(1-y_{i}\right)\log\left(1-p_{i}\right)\right). \end{array}$$

The optimizer of the model is the adaptive moment estimation optimizer Adam [37]. In Adam, the first and second moments of the gradient are used to update and correct the current learning rate [29]. During the training process, the parameters of the Adam optimizer are set to a learning rate of 0.01 and the maximum number of epochs to 50 and leave other parameters as default. If there is no improvement for more than 5 epochs, and the minimum learning rate is set to 0, the learning rate will drop by 0.5 times. The network will continue to be trained at this learning rate until the loss of the network no longer changes significantly. At this time, the learning rate will be reduced again, until the maximum number of iterations is reached. The batch size is 8, and the coordinates of the input point cloud are normalized to between 0 and 1.

To evaluate the effect of the model, this paper uses accuracy and intersection over union (IoU, intersection over union [5]) as evaluation indicators, see formulas (3) and (4). Among them, TP, FP, and FN represent real samples, false-positive samples, and false negatives. This article uses 10% of the total data set as the test set.

(3) $$\begin{array}{c} \text{Acc}=\frac{1}{m}\overset{m}{\underset{i=1}{\sum }}f_{i},\;f_{i}=\left\{\begin{array}{c} 1\;y_{i}=p_{i,}\\ \begin{array}{cc} 0 & y_{i}\neq p_{i} \end{array}, \end{array}\right. \end{array}$$

(4) $$\begin{array}{c} \text{IoU}=\frac{\text{TP}}{\text{FP}+\text{TP}+\text{FN}}. \end{array}$$

## 3. Results

**Figure 7:**
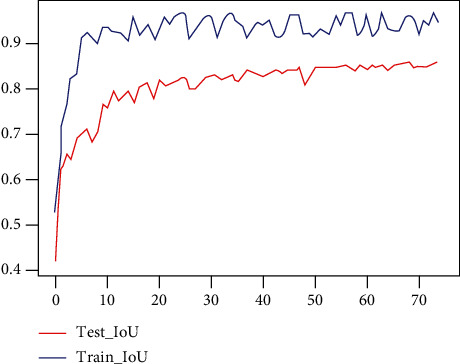
IOU vs. epoch curve.

This article builds the above neural network model based on Tensorflow and uses a single NVIDIA GeForce GTX 1080 GPU to train for 8 hours on the GPU server of Ubuntu 16.04 to complete the experiment. The IoU changes during training are shown in Figure 7.

**Figure 8:**
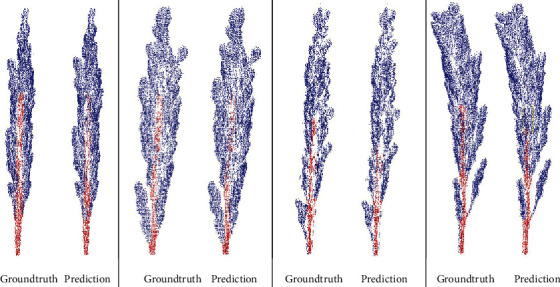
The segmentation results.

The training results show that the designed and optimized neural network model can effectively segment the rice ear point cloud. The final IoU reached 0.86, and the accuracy rate reached 0.93. The segmentation results are shown in Figure 8.

### 3.1. Ablation Experiment

The Panicle-3D network proposed in this article is inspired by U-Net and ResNet. Drozdzal et al.'s research on the importance of long-short jumps in segmentation tasks further confirms the effectiveness of the long-short jump structure [28]. From the structural point of view, the large-scale crosslayer connection structure of U-net and the small scale crosslayer connection structure of ResNet are called long-jump structure and short-jump structure, as shown Figures 5 and 6.

We did ablation experiments in three situations, which were to turn on long jumps and short jumps, turn on long jumps and turn off short jumps, and turn on short jumps and turn off long jumps. And we still use the crossentropy loss (cost) function in Equation (2).The training loss and validation loss are shown in Table 1. Through experimental comparison, we found that the model that uses both long and short jumps has the lowest loss.

### 3.2. Network Model Comparison Experiment

**Figure 9:**
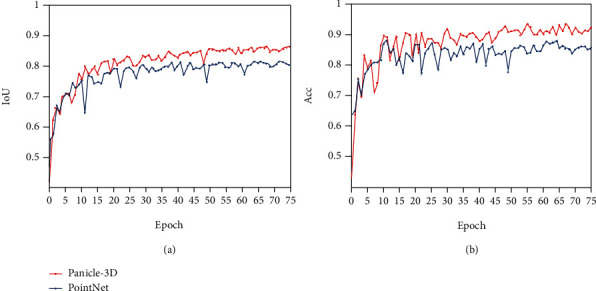
The comparison of Panicle-3D and PointNet (a) IoU vs. epoch (b) Acc vs. epoch.

To verify the effectiveness of the network, the PointNet network [15] is used to train the data set. PointNet is also an end-to-end model that uses discrete point clouds as direct input and directly outputs the predicted probability of each point. The comparison of IoU and accuracy of the two networks is shown in Figure 9:

To compare the results, see Table 2. The results show that the method in this paper is significantly better than the PointNet model in terms of IoU and accuracy.

## 4. Discussion

The point cloud segmentation of plant organs is of great significance in the automated measurement of plant phenotypic parameters. In this paper, firstly, the three-dimensional point cloud scanning device based on the DLP4500 module and automatic turntable is designed to collect rice point cloud data. After data preprocessing and annotation, a data set containing 200 rice point clouds is obtained. Then, for the plant point cloud that is difficult to segment by traditional machine learning methods, a 3D point cloud convolutional neural network architecture is introduced, and a fast and effective point cloud segmentation method is designed based on this architecture to achieve high precision of the rice ear point cloud segmentation. The segmentation accuracy rate reached 93.4%, and the IoU reached 86.1%, both of which are better than the classic point cloud processing model PointNet. The experimental results show that the Panicle-3D proposed in this paper outperforms its counterparts in the segmentation of the plant point cloud.

Notably, as a deep learning method, Panicle-3D also has certain limitations. The performance of Panicle-3D is positively related to the volume of the data set, and the collection and labeling of point cloud data are very labor-intensive. In addition, when the plant organs are wrapped in leaves, humans often cannot label the point cloud data set correctly. To obtain the ground truth of the hidden stem, a destructive means is to detach the grains from the panicle when making the data set.

Based on such a hardware platform and a deep neural network segmentation method, we can obtain the precise segmentation of the stems and ears of the rice point cloud. Thus, we can obtain the overall phenotypic parameters of the rice ear, such as the diameter of the stem, the length of the stem, the length, height, and width of the panicle, the geometric characteristics of the main panicle and spikelet, and the distribution of seedlings in the ear.

Compared with traditional phenotypic parameter measurement methods, the method proposed in this study helps to achieve automation of crop phenotypic parameters and provides support for functional genetic analysis and breeding.

## Figures and Tables

**Table 1:** Comparison between different connection methods.

Method	Training loss	Validation loss
Long and shot skip connections	0.162	0.164
Only short skip connections	0.186	0.201
Only long skip connections	0.206	0.191

**Table 2:** Comparison of common plant 3D reconstruction methods.

Network	Acc	IoU	Epochs
Panicle-3D	0.93	0.86	50
PointNet	0.90	0.84	50

## Data Availability

The Python code of the point cloud dataset and model is available on Github at https://github.com/gitDux/Panicle-3D.

## Supplementary Materials

Supplementary Materials The supplementary materials include physical images of equipment and point cloud dataset images. S1: photos of point cloud collection equipment S2: some point cloud data in the panicle of rice point cloud dataset.
