# Supplementary material for: Panicle-3D: Efficient Phenotyping Tool for Precise Semantic Segmentation of Rice Panicle Point Cloud
Source: Plant Phenomics. 2021 Dec 23;2021:9838929. doi: 10.34133/2021/9838929 (PMC8720256; doi:10.34133/2021/9838929)
Supplement: Supplementary Materials — The supplementary materials include physical images of equipment and point cloud dataset images. S1: photos of point cloud collection equipment S2: some point cloud data in the panicle of rice point cloud dataset. [file 9838929.f1.docx]

Supplementary Materials

The supplementary materials include physical images of equipment and point cloud dataset images.

**S1. Photos of point cloud collection equipment**

Figure S1 is a photo of a point cloud collection device specially built to standardize the point cloud image collection of rice panicles.

**
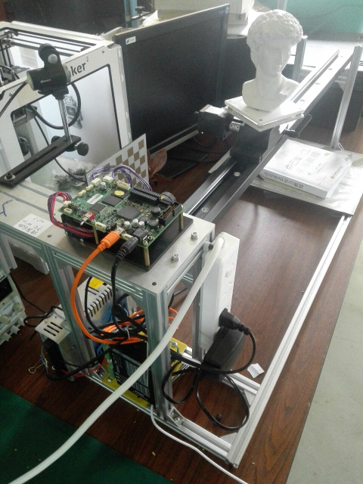

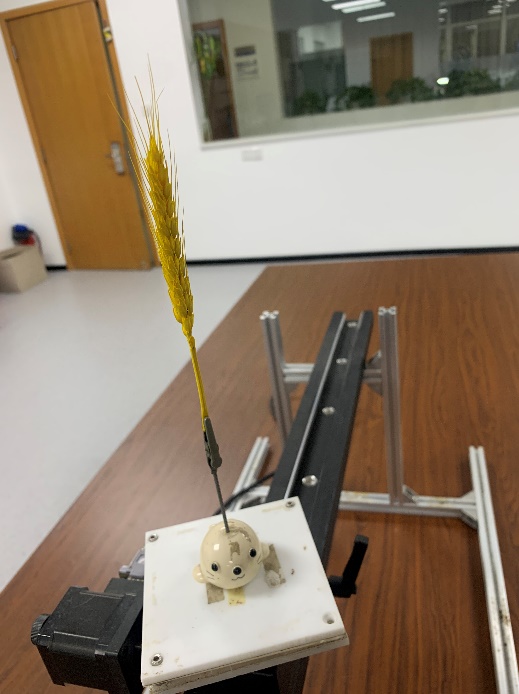
**

Figure S1. Photos of point cloud collection equipment

**S2. Some point cloud data in the panicle of rice point cloud dataset**

Figure S2 shows some point clouds in the rice panicle point cloud dataset.

| 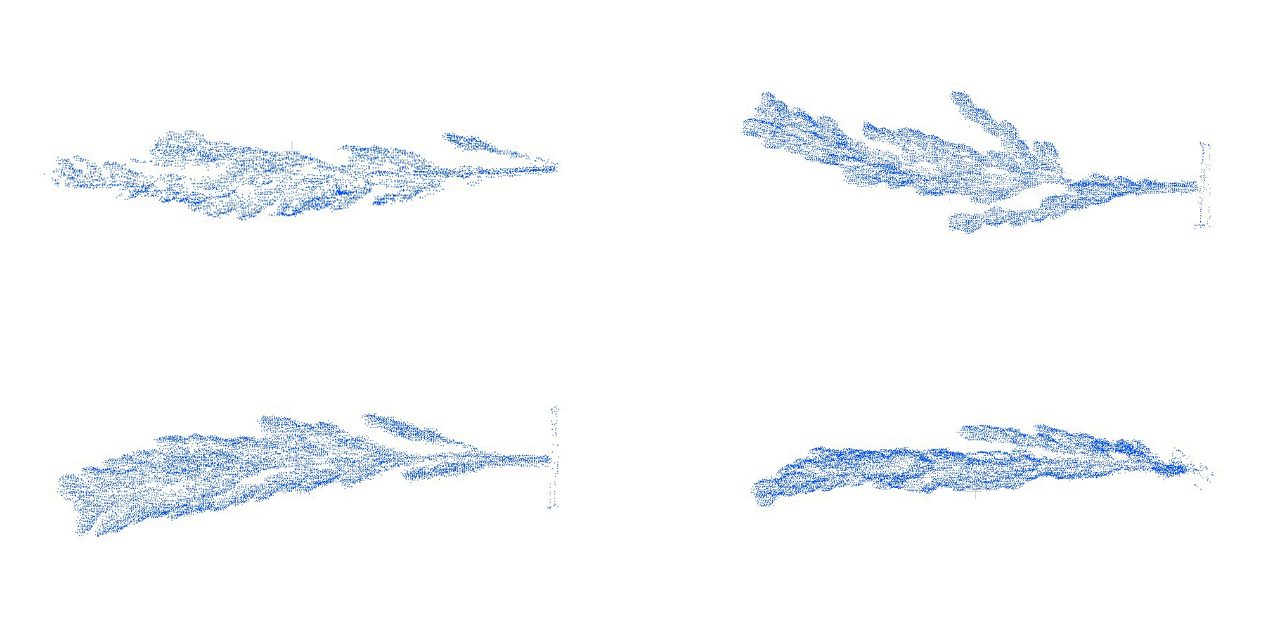  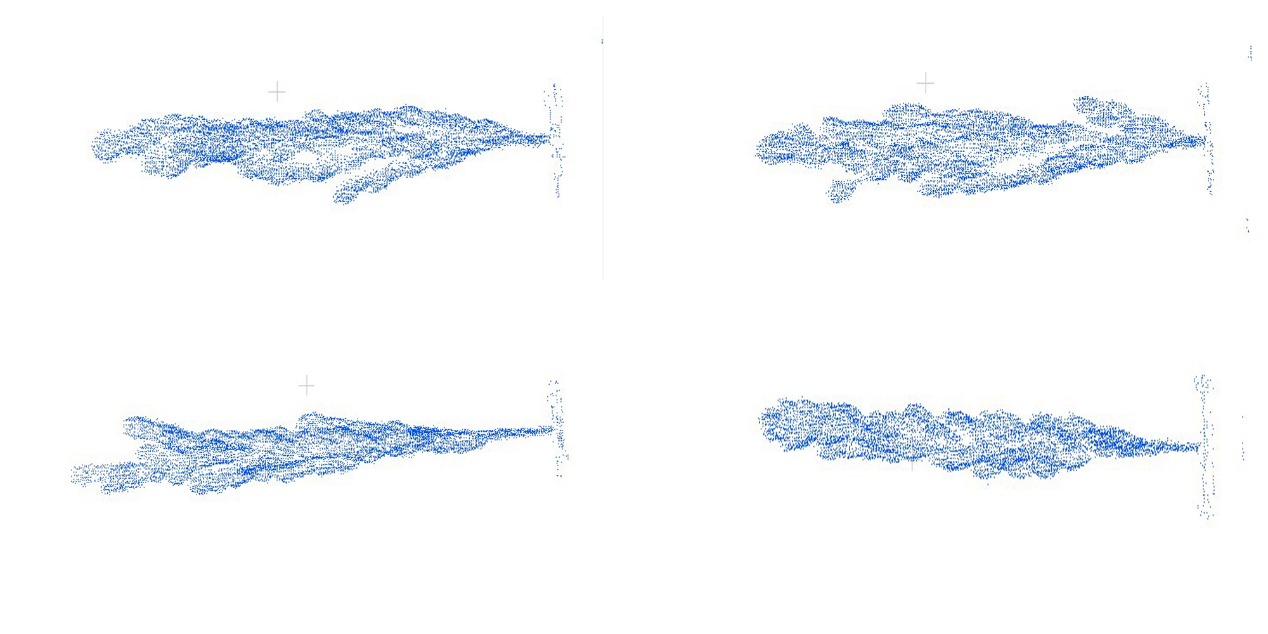  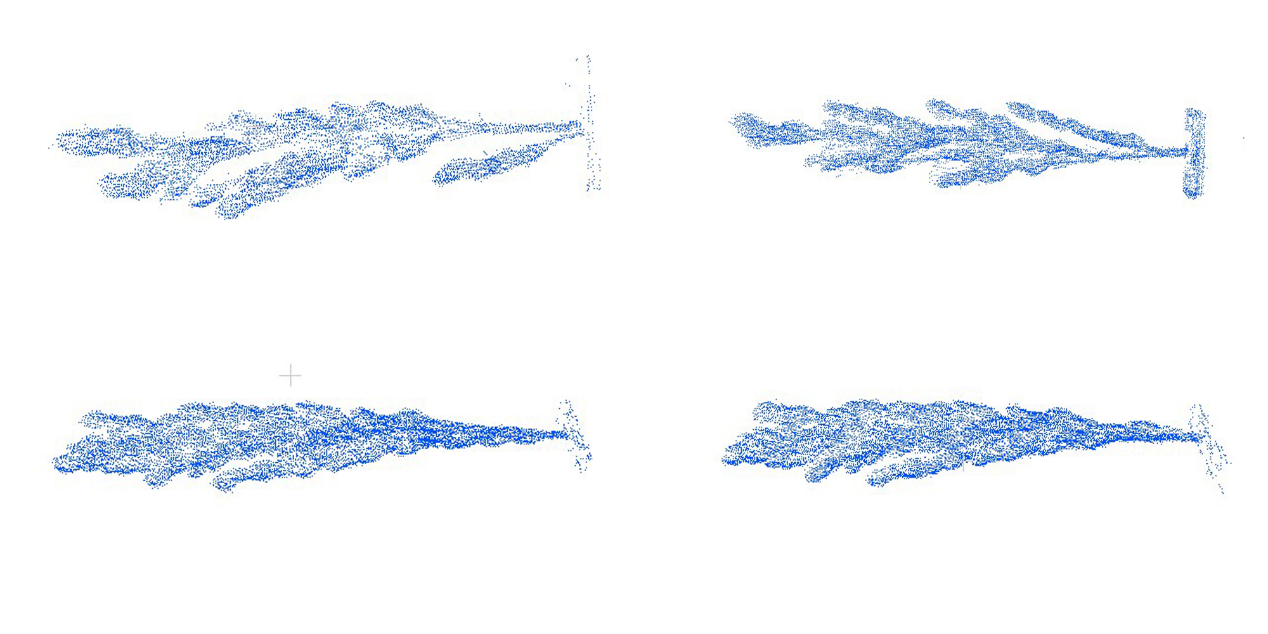  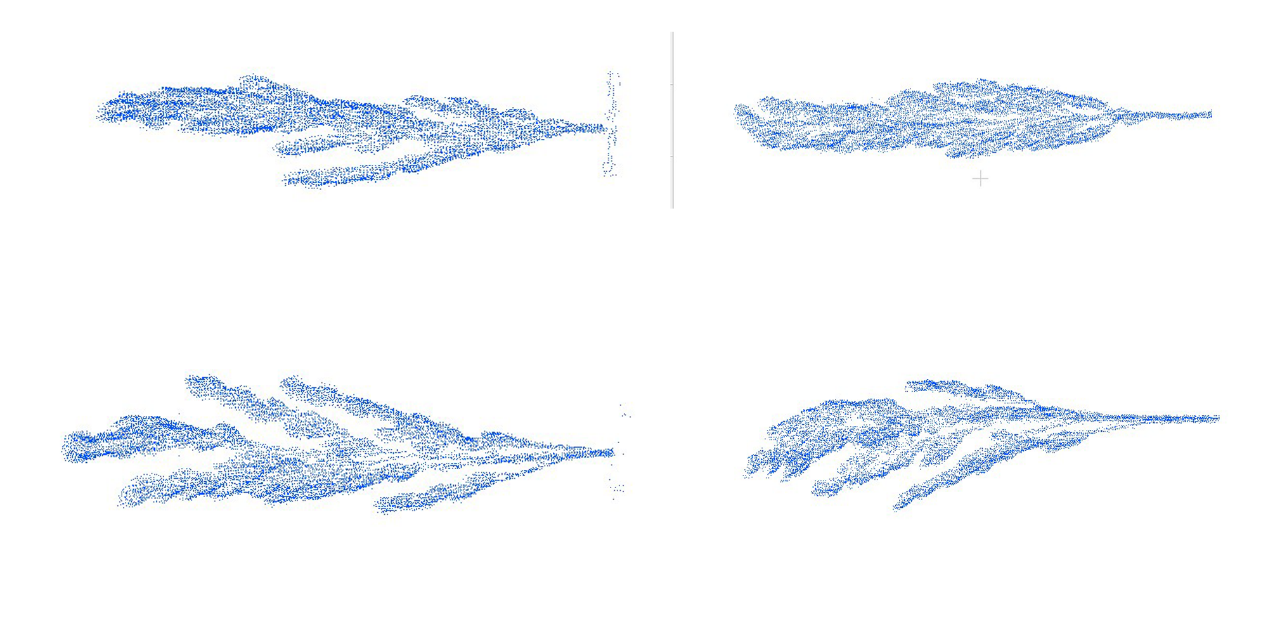  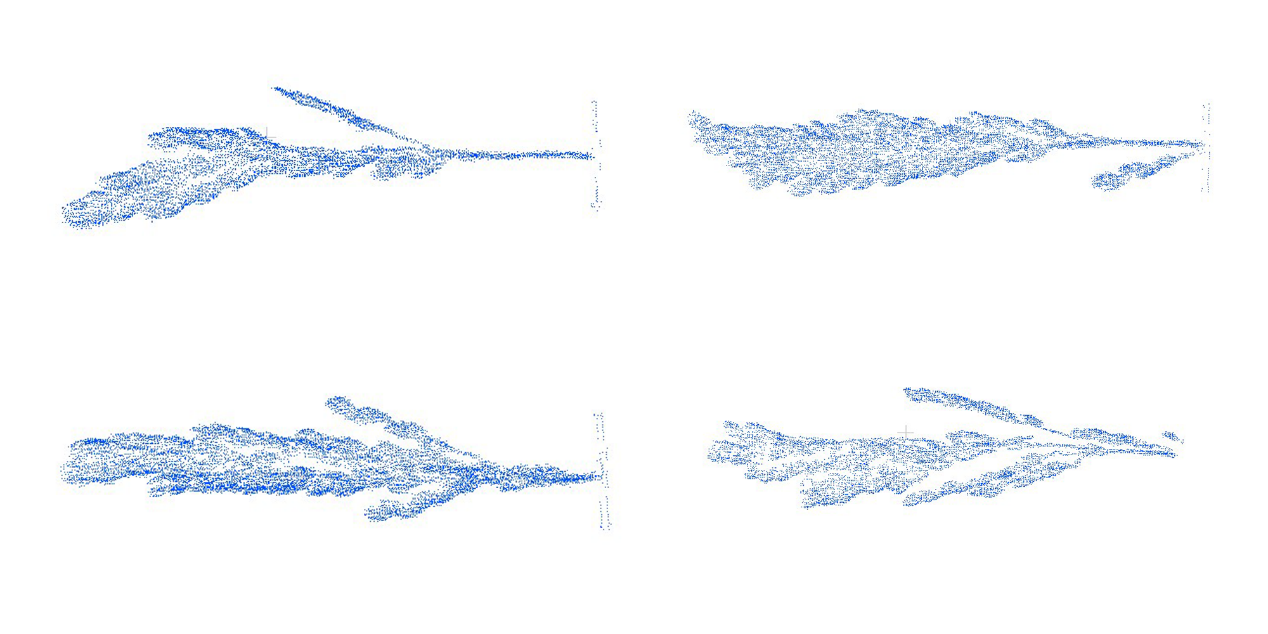 |
| --- |

Figure S2. Point clouds dataset
